# Preparation, Structural Characterization, and Property Investigation of Gallic Acid-Grafted Fungal Chitosan Conjugate

**DOI:** 10.3390/jof7100812

**Published:** 2021-09-28

**Authors:** Weslley Souza Paiva, Moacir Fernandes Queiroz, Diego Araujo Sabry, André Luiz Cabral Monteiro Azevedo Santiago, Guilherme Lanzi Sassaki, Anabelle Camarotti Lima Batista, Hugo Alexandre Oliveira Rocha

**Affiliations:** 1 Postgraduate Programe in Biotechnology (RENORBIO), Federal University of Rio Grande do Norte (UFRN), Natal 59078-970, RN, Brazil; 2 Biomedicine Departament, Medical Sciences College, Potiguar University (UNP), Natal 59056-000, RN, Brazil; moacirfqn@gmail.com; 3 Laboratorio de Biotecnologia de Polímeros Naturais-BIOPOL, Departament of Biochemistry, Federal University of Rio Grande do Norte (UFRN), Natal 59078-970, RN, Brazil; popoh.diego@gmail.com; 4 Departament of Micology, Federal University of Pernambuco (UFPE), Recife 50670-901, PE, Brazil; andre.masantiago@ufpe.br; 5 Department of Biochemistry and Molecular Biology, Federal University of Paraná, Curitiba 81531-980, PR, Brazil; sassaki@ufpr.br; 6 Department of Agriculture, Federal University of Paraíba (UFPB), Bananeiras 58220-000, PB, Brazil; bellecamarotti@gmail.com

**Keywords:** oxidative stress, *Rhizopus arrhizus*, fungal chitosan, antioxidant, gallic acid

## Abstract

Oxidative stress is the cause of numerous diseases in humans; therefore, there has been a continuous search for novel antioxidant molecules. Fungal chitosan is an attractive molecule that has several applications (antifungal, antibacterial, anticancer and antiparasitic action) owing to its unique characteristics; however, it exhibits low antioxidant activity. The aim of this study was to obtain fungal chitosan (Chit-F) from the fungus *Rhizopus arrhizus* and synthesize its derivative, fungal chitosan-gallic acid (Chit-FGal), as a novel antioxidant chitosan derivative for biomedical use. A low molecular weight Chi-F (~3.0 kDa) with a degree of deacetylation of 86% was obtained from this fungus. Chit-FGal (3.0 kDa) was synthesized by an efficient free radical-mediated method using hydrogen peroxide (H_2_O_2_) and ascorbic acid. Both Chit-F and Chit-FGal showed similar copper chelating activities; however, Chit-FGal was more efficient as an antioxidant, exhibiting twice the total antioxidant capacity than Chi-F (*p* < 0.05). Furthermore, H_2_O_2_ (0.06 M) promoted a 50% decrease in the viabilities of the 3T3 fibroblast cells. However, this effect was abolished in the presence of Chit-FGal (0.05–0.25 mg/mL), indicating that Chit-FGal protected the cells from oxidative damage. These results suggest that Chit-FGal may be a promising agent to combat oxidative stress.

## 1. Introduction

In the human body, an imbalance between the oxidant and antioxidant molecules promotes the formation of reactive species that give rise to a state known as “oxidative stress”, which favors oxidative damage in macromolecules and cells and can even cause cell death [1,2]. Among the reactive species, the main ones associated with oxidative stress are the reactive oxygen species (ROS) [3].

Living organisms have finely regulated systems to maintain low levels of ROS, that is, their production and elimination processes are well balanced, which results in the maintenance of steady levels of ROS in the body. However, this balance can be affected under certain circumstances. Several factors may result in an imbalance of ROS levels, such as the increase in the number of compounds that undergo auto-oxidation, and inactivation of antioxidant enzymes. The consequences of this increase in the number of compounds that undergo auto-oxidation differ depending on the levels of ROS, location of ROS generation, and efficiency of antioxidant systems as well as the cellular targets they interact with [4].

The imbalance in the regulation of ROS levels can cause several diseases, such as epilepsy [5,6], lung diseases [7], cancer [8], diabetes [9,10], Parkinson’s disease [11,12], and Alzheimer’s disease [13,14]. Therefore, there is a constant search for novel antioxidant molecules that can help to regulate this system. Among all the studied molecules, chitosan appears to be one of the most promising agents for the regulation of ROS levels [15,16,17].

Chitosan is a biopolymer formed by the deacetylation of some glucopyranose residues from chitin [18]. It is composed of 2-acetamido-2-deoxy-d-glucopyranose and 2-amino-2-deoxy-d-glucopyranose units. However, a molecule is only considered to be chitosan when more than 50% of the polymer residues are 2-amino-2-deoxy-d-glucopyranose units [19]. Chemical deacetylation of the chitin obtained from crustaceans is the best-known method to obtain chitosan [20]. Some species of fungi naturally synthesize chitosan, mainly the species belonging to the class Zygomycetes, and it can be extracted from their cell walls [21]. Therefore, the terms “fungal chitosan” and “animal chitosan” are used to differentiate the two sources of chitosan [22].

Animal chitosan is the most commercially used type of chitosan owing to the high yield obtained from its extraction process, which is approximately 2–3 times greater than that of the fungal chitosan. However, during the processing and purification of animal chitosan, large amounts of chemical reagents are used, which increase the production cost and lower the yield of the extracted chitosan [23,24]. In addition, animal chitosan depends on seasonal factors in the region from where the crustacean is obtained, which makes it difficult to standardize its implementation on an industrial scale [25]. The allergenicity of some proteins such as tropomyosin and arginine kinase, which accumulate residually in the chitosan molecule when it is not purified correctly, limit the use of these chitosan molecules in the food and medical industries [26,27,28]. The deproteinization processes of animal chitosan require the use of strong acids and bases, which increase the production cost and cause environmental damage owing to the generation of a large amount of industrial waste [29]. These types of problems are not reported during the extraction of fungal chitosan [24].

Fungal chitosan is already being produced and marketed by several companies in different countries, such as Belgium, Canada, and the United States, for medical and/or pharmaceutical applications [30]. Several studies have demonstrated the potential of fungal chitosan to act as an antifungal [31], antibacterial [32], antiparasitic [33,34], and anticancer agent [35]. However, despite the ecological advantages associated with its production as well as its non-allergenic nature, fungal chitosan is not used more widely than animal chitosan because of its lower yield. Therefore, efforts have been made to increase its consumption, discover novel species of chitosan-producing fungi, and increase the productivity, extraction, purification, and identification of novel types of fungal chitosan and their derivatives.

Within this context, one potential candidate is the fungus *Rhizopus arrhizus*. *R. arrhizus* produces significantly higher amounts of chitosan than other fungi, such as *Aspergillus niger*, *Zygosaccharomyces rouxii*, and *Candida albicans* [36]. In addition, chitosan from this fungus has a much smaller molecular mass (~5.2 kDa) [37] than that from other fungi, which suggests that it may exhibit differential activities from other chitosan molecules. The fungi from Zygomycetes (same class as *R. arrhizus*) are known for being easy-to-handle, fast-growing on different substrates, and resistant to extreme environmental conditions [38,39,40]. These unset characteristics make *R. arrhizus* chitosan a promising candidate for different types of studies.

However, the antioxidant activity of fungal chitosan has not yet been evaluated. Queiroz et al. (2019) demonstrated that it is possible to increase the antioxidant activity of animal chitosan by conjugating it with gallic acid (GA) [41]. Therefore, this study aimed to obtain fungal chitosan from *R. arrhizus*, conjugate it with GA, and evaluate the antioxidant activities of the native and modified chitosan molecules to understand their potential to combat oxidative stress.

## 2. Materials and Methods

### 2.1. Procuring the Fungal Strain and Production of Chitosan

**Figure 1:**
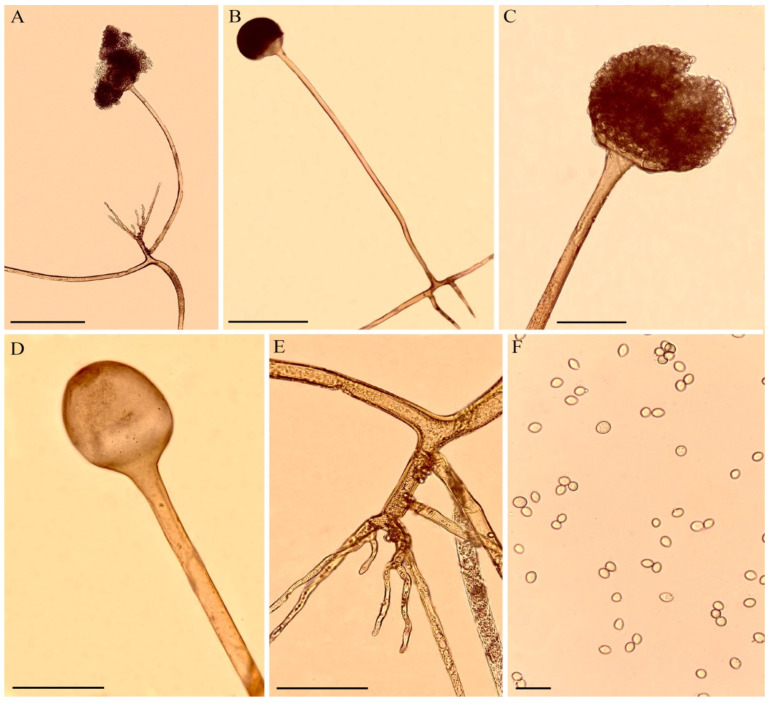
Morphology of *R. arrhizus*. (**A**,**B**). Sporangiophore with sporangium and rhizoid. (**C**). Sporangiophore with sporangium. (**D**). Sporangiophore with columella. (**E**). Rhizoid. (**F**). Sporangiospores. Scale bars: (**A**,**B**) = 100 μm, (**C**–**E**) = 50 μm, (**F**) = 20 μm.

Soil samples were collected from 10 different points in the Estação Ecológica do Sérido reserve, under the license number 36672-1 SISBIO/ICMBio. This reserve is in the southwest region of the state of Rio Grande do Norte, Brazil (06°35′ S and 06°40′ S; 37°20′ W and 37°39′ W). This conservation unit is in the caatinga biome, a region with high temperatures and low rainfall, with a total extension of 1123.59 hectares, exhibiting vast environmental diversity. The sampling areas were randomly selected and marked by the Global Positioning System (GPS). The isolation was performed using the Sabouraud agar medium (KASVI, São José dos Pinhais, PR, Brazil) and incubated in an oven at 28 °C, according to a previously described method [42]. Figure 1 shows several structures of the fungus *R. arrhizus.*

### 2.2. Molecular Identification

Genomic DNA was extracted from the mycelium growing on the potato dextrose agar (PDA) at 28 °C for 72 h using the cetyltrimethylammonium bromide (CTAB) DNA Extraction Protocol, as adapted from previously described methods [43]. The ribosomal DNA ITS1-5.8S-ITS2 and large subunit (LSU) regions were amplified by polymerase chain reaction (PCR) on a Peltier PTC100^®^ Thermocycler (MJ Research, Inc., Hampton, NH, USA) in a total volume of 25 μL of the sample. The rDNA internal transcribed spacer (ITS) regions were amplified using the primers, ITS1 (5′-TCCGTAGGTGAACCTGCGG-3′) and ITS2 (3′-GCTGCGTTCTTCATGAGC-5′) [44]. The D1/D2 LSU region of the rDNA was sequenced using primers NL1 (5′-GCATATCAATAAGCGGAGGA-3′) and NL4 (5′-GGTCCGTGTTTCAAGACGGGTCG-3′). The amplicons were purified using the PureLink-PCR Purification Kit C/50rxn Columns (Invitrogen, Waltham, MA, USA) and sequenced by ACT Gene Molecular Analyses (Alvorada, RS, Brazil) [45]. The identified copies were then cataloged into the Culture Bank of the Federal University of Pernambuco, Recife, PE, Brazil and kept in solid PDA medium at pH 5.5 (N° URM 8111).

### 2.3. Chitosan Extraction

After isolating and identifying the strains, we performed the chitosan production test. *R. arrhizus*. The spores were collected and stored in 15 mL of sterile distilled water (standard solution). Approximately 10^5^ spores/mL of the standard solution were then added to 400 mL of the yeast extract peptone dextrose (YPD) medium (Yeast Extract 10 g; Peptone 20 g; Dextrose 20 g per liter) and incubated at 28 °C for 96 h in a static mode. The biomass was filtered and lyophilized, and chitosan was extracted according to the previously described method [46], with some modifications [47]. The sample was named Chit-F.

### 2.4. Physicochemical Characterization of the Chitosan Molecule

#### 2.4.1. Determination of Molecular Weight

The apparent molecular weight of samples was determined by HPLC (High-Performance Liquid Chromatography) Accela (Thermo scientific, Waltham, MA, USA) com coluna Waters ultragel 250 (EUA, Milford, MA, USA) acoplada. The samples were eluted with sodium nitrite 0.1 M, at a flow rate of 1 mL/min 30 °C for10 min. The column was calibrated using different dextrans (3; 6; 40; 70 and 100 kDa) purchased from Sigma (St. Louis, MO, USA).

#### 2.4.2. Measurement of the Protein and Phenolic Compounds

The protein content was quantified using the Coomassie brilliant blue reagent and bovine serum albumin as previously described [48]. The content of the phenolic compounds was measured by the Folin–Ciocalteu reagent as previously described [49]. The Folin–Ciocalteu procedure was used to evaluate the GA fungal chitosan.

#### 2.4.3. Conjugation of GA and Chit-F

First, 500 mg of Chit-F was dissolved into 10 mg/mL of acetic acid in water solution (2% *v*/*v*). Then, 1 mL of 1 M hydrogen peroxide (H_2_O_2_) and 0.054 g ascorbic acid were added to this solution. After 30 min, 1.4 mmol GA was introduced to the reaction and incubated for 24 h, at room temperature. The solution was then centrifuged using Amicon^®^ Ultra-15 centrifugal filter (Millipore, Burlington, MA, USA) with 3 kDa cut-off until all unreacted GA was removed [41,50]. The GA conjugated Chit-F solution was named “Chit-FGal,” and it was frozen and lyophilized until future use.

#### 2.4.4. Fourier Transformed Infrared Spectroscopy (FTIR) and Degree of Deacetylation (DD)

A Nexus 470 ESP FTIR spectrometer (Thermo Nicolet, Madison, WI, USA) was used to obtain the infrared spectra between 500 and 4000 cm^−1^ of a tablet containing potassium bromide (KBr) mixed with different samples (5 mg). Thirty-two scans at a resolution of 4 cm^−1^ were evaluated and referenced against air. DD was determined using Equation (1):

DD (%) = 100 − [(A1655/A3450) × 115](1)

#### 2.4.5. Nuclear Magnetic Resonance (NMR) Spectroscopy

The samples (50 mg) were dissolved in 800 µL of acidified deuterium oxide (D_2_O). NMR spectra (^1^H) were obtained in a Bruker Avance III HD 600 MHz spectrometer (Bruker BioSpin Corporation, Billerica, MA, USA) equipped with a 5 mm inverse quadruple resonance probe (QXI) at 70 °C. The chemical shifts were expressed in δ relative to trimethylsilylpropanoic acid (TMSP) at δ = 0.00, in accordance with the International Union of Pure and Applied Chemistry (IUPAC) recommendations.

#### 2.4.6. Scanning Electron Microscopy (SEM) Analysis

The chitosan samples were processed and analyzed by SEM (Shimadzu Electron microscope, model SSX550; Shimadzu Corp., Kyoto, Japan). Briefly, 20 μL of each chitosan (0.5 mg/mL) sample was loaded onto a carbon-coated copper grid without gold coating and air-dried for 10 min under vacuum. The grid chamber was then placed in the SEM room and incubated in the dark at 10–20 °C for 2 h. Representative images of each of the three independent experiments are shown.

### 2.5. Antioxidant Activity

#### 2.5.1. Copper Chelation

The copper chelation test was performed as described previously [51]. Briefly, 96-well microplates with a reaction mixture containing different concentrations of samples (0.1–2 mg/mL), pyrocatechol violet (4 mM) and copper II sulfate pentahydrate (50 mg/mL) were homogenized with the aid of a micropipette, and the absorbance of the solution was measured at 632 nm using a microplate reader (SpectraMax^®^ M2/M2e, Molecular Devices, São José, CA, USA).

#### 2.5.2. Determination of Total Antioxidant Capacity

The determination of the total antioxidant capacity assay was carried out as described previously [52]. It is based on the reduction of molybdenum (Mo) (VI) to Mo (V) by the sample and the subsequent formation of a phosphate green complex/Mo (V) at an acidic pH. Tubes containing the chitosan and reagent solution (0.6 M sulfuric acid, 28 mM sodium phosphate, and 4 mM ammonium molybdate) were incubated at 95 °C for 90 min. After the mixture had cooled to room temperature, the absorbance of each solution was measured at 695 nm using a microplate reader (SpectraMax^®^ M2/M2e, Molecular Devices, São José, CA, USA). Total antioxidant capacity was expressed as the ascorbic acid equivalent.

### 2.6. Cell Culture Experiments

#### 2.6.1. Cytotoxicity Assay

The cytotoxicity assay was performed using the 3-(4,5-dimethylthiazol-2-yl)-2,5-diphenyltetrazolium bromide (MTT) method as previously described [53]. The fibroblast cells (3T3 ATCC CCL-92) were cultured in culture flasks in the Dulbecco’s modified Eagle medium (DMEM) medium with 10% (*v*/*v*) fetal bovine serum (FBS), 100 μg/mL streptomycin, and 100 IU/mL penicillin (Sigma, St. Louis, MO, USA). The cells were cultured in sterile 96-well plates at a density of 5 × 10^3^ cells/well and incubated at 37 °C and 5% carbon dioxide (CO_2_) for 24 h. After this period, the medium was exchanged with serum-free DMEM to synchronize the cells in the G_0_ phase (active cells do not enter into any cell cycle stage). The cells were kept in this medium for 24 h. Afterward, the medium was removed and another medium containing 10% FBS and different concentrations of the samples (50, 100, 250, and 500 μg/mL) were added. After 24 h of incubation, the cell traces were removed by washing the cells with phosphate-buffered saline (PBS). Then, a serum-free culture medium containing 12 mM MTT (Sigma, St. Louis, MO, USA) was added to the samples to determine the ability of the cells to reduce MTT. The cells were then incubated at 37 °C and 5% CO_2_ for 4 h. To solubilize the reduced MTT product, 100 µL of ethyl alcohol was added to each well and thoroughly mixed using a multichannel pipettor. After 15 min of the addition of alcohol, the absorbance at 570 nm was read using a microplate reader (Thermo Labsystems, Franklin, MA, USA).

#### 2.6.2. Induced Oxidative Stress Assay

The 3T3 fibroblast cells (1 × 10^6^ cells/mL) were plated in 6-well plates in the presence of DMEM supplemented with 10% FBS. After 24 h, these plates were washed with PBS (Phosphate-Buffered Saline) and the DMEM medium supplemented with FBS (10%) containing Chit-F and Chit-FGal (50, 100, and 250 μg/mL) was added. Then, 0.06 mM H_2_O_2_ was added to the wells to induce oxidative stress. The plates were kept under growth-inducive conditions (37 °C, 5% CO_2_, protected from light) for 6 h. Then, the medium was replaced with the DMEM medium with 10% FBS. After 24 h, the cells were evaluated by the MTT assay as previously described [54].

#### 2.6.3. Nuclear Morphology

The 3T3 cells were subjected to the same experimental conditions as described in Section 2.4.2. After, the cells were washed with PBS and fixed with 4% paraformaldehyde in PBS for 30 min at room temperature. After washing twice with PBS, cells were maintained in PBS containing 0.1% Triton X-100 at room temperature for 30 min. Fixed cells were washed with PBS and stained with DAPI (4′,6-diamidino-2-phenylindole) (1 μg/mL) solution for 30 min at room temperature. Nuclear morphology of cells nuclei was examined under a fluorescent microscope (TE-Eclipse 300, Nikon, Melville, NY, USA). Data presented are representative of those obtained in at least three independent experiments carried out. 

DAPI staining was also used to quantify cell nuclei (normal/condensed/fragmented). Images were captured from ten different fields and about 100 cells were counted for each field on UV microscopy and analyzed using the NIS-Elements AR analysis software version 4.00.03 (Nikon Instruments Inc., Melville, NY, USA, 2011). Counting was carried out in three independent experiments for each sample.

### 2.7. Statistical Analysis

At least three independent experiments were performed for each test. All data are presented as the mean ± standard deviation (n = 3). The analysis of variance (ANOVA) test was performed to check the difference between the results. The Student–Newman–Keuls test (*p* < 0.05) was used to determine the similarities found by ANOVA. All the tests were performed using the GraphPad Prism 5 software (GraphPad Softwares, San Diego, CA, USA).

## 3. Results and Discussion

### 3.1. Obtaining Fungal Chitosan and Determining Its Yield

The yield of chitosan obtained from the dry biomass of *R. arrhizus* was 22.53 mg of chitosan/g of fungus. This value is similar to the yield (26 mg/g) of chitosan obtained from the dry masses of the fungi, *Syncephalastrum racemosum* [55] and *Pleurotus ostreatus* (24 mg/g) [56], but lower than the yield (37.7 mg/g) obtained from the fungus, *Agaricus bisporus* [56].

However, it is possible to increase the amount of chitosan obtained from a specific fungal species by changing the composition of its growth medium. Berger et al. (2020) cultivated two species of fungi, *Cunninghamella elegans* UCP 1306 and *Cunninghamella phaeospora* UCP 1303, in different media containing different percentages of cashew apple juice (CAJ) and cheese whey (CW) [57]. After the extraction of chitosan, they observed that the two fungi produced a greater amount of chitosan in the presence of higher levels of CAJ and CW. *C. elegans* UCP 1306 was more efficient as the yield of chitosan obtained from it ranged from 6.9 mg/g (in a medium without any additives) to 64.4 mg/g (in the presence of 40% (*v*/*v*) of CAJ and 30% (*v*/*v*) of CW).

There was only one other study that determined the yield of chitosan extracted from *R. arrhizus* [24]. In that study, the authors obtained a chitosan yield of 29.3 mg/g, which is slightly higher than the yield obtained in our study. However, in this study, the fungi grew in a medium enriched with steep liquor and honey. These data indicate that *R. arrhizus* can produce greater amounts of chitosan depending on the composition of the medium in which it is grown. Therefore, future studies can evaluate the yield of chitosan obtained from this fungus when cultivated in different media.

### 3.2. Chitosan Characterization

#### 3.2.1. Degree of Deacetylation (DD)

The DD% is a crucial property of chitosan, which indicates the number of amine groups in the molecule, especially on the second carbon. The greater the amount of amine, the greater the range of applications and the possibility of chemical modifications that can be performed in chitosan [58,59]. The DD found in the chitosan extracted from *R. arrhizus* was 85.66% ± 0.57, which classifies this molecule as a chitosan suitable for biomedical use, as the chitosan with DD ranging from 75–85% is considered to be good for medical applications [60]. The *R. arrhizus* chitosan DD value described here was higher than that presented by [57] for the chitosan obtained from the same fungus, in which case the DD was equal to 70.5%. This difference in both studies was probably due to the cultivation of fungi in different media. The DD described here is similar to the values described for the chitosan extracted from other fungi of the genus *Rhizopus*, including the DD of 85.2% of the chitosan extracted from *Rhizopus stolonifer* [61] and DD of 86.2% of the chitosan extracted from the fungus *Rhizopus oryzae* [62]. This indicates that chitosan from fungi of the *Rhizopus* genus has a DD that gives them the potential to be used for biomedical applications. However, further studies with chitosan from other fungal species of the genus *Rhizopus* need to be performed to confirm this observation.

#### 3.2.2. Quantification of Proteins, Phenolic Compounds, Apparent Molecular Weights, and Yield of Chemically Modified Chitosan with GA

The data presented above and the infrared and NMR analysis (Section 3.2.3 and Section 3.2.4, respectively) confirmed that the compound obtained from *R. arrhizus* was chitosan. Then, this chitosan was conjugated with GA as described in the methods section.

**Figure 2:**
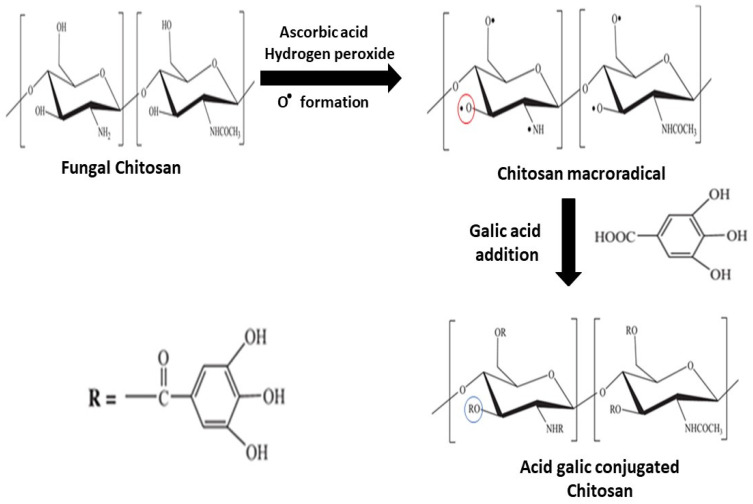
Scheme of the conjugation process of gallic acid (GA) and fungal chitosan (Chit-F). Step 1 is the addition of the redox pair and the formation of macroradicals. The red circle shows the radical. Step 2 is the addition of GA to the solution and the formation of the conjugated molecule. The blue circle shows the position of linked gallic acid. R—GA or hydrogen.

Figure 2 shows the main steps in the synthesis of Chit-FGal. In aqueous media, ascorbic acid is oxidized by peroxide, forming ascorbate and hydroxyl radical. The last one starts attacking Chit-F leading to the formation of macroradicals, which are chitosan molecules that have regions with unpaired electrons (therefore, they are also reactive species) that act as entry points for GA [50]. The second step is the addition of GA in the reaction. In this step, a carboxyl group present in GA reacts with the entry points in the macroradicals, leading to the formation of a covalent bond and synthesis of Chit-FGal.

The conjugation of phenolic compounds to polysaccharides was first reported by Domnina et al. [63]. However, a simple, efficient, and environment-friendly method to perform this type of conjugation was only developed in 2009 [50]. This method is very efficient and environmentally friendly as it does not induce the generation of any toxic waste, only producing ascorbic acid, H_2_O_2_, and GA. Furthermore, this method only has a few steps, making it a feasible alternative to other polysaccharide modification methods [50]. Therefore, it was the method chosen in this study for the conjugation of Chit-F with GA.

After the conjugation with GA, the fungal chitosan was called Chit-F, and the chitosan conjugated with GA was called Chit-FGal. We analyzed the two molecules for their content of phenolic compounds and proteins (See Table 1).

The presence of proteins was not detected in any of the two evaluated samples (Chit-F and Chit-FGal), which shows that chitosans are free of this contaminant. Phenolic compounds were not detected in Chit-F, but approximately 4% was found in Chit-FGal, which is four times more than that in Chit-F, thereby indicating the success of the conjugation process with GA.

The apparent molecular mass of the two samples was also determined (Table 1). Chit-F had an apparent molecular mass of 3 kDa. This value resembles that of the *R. arrhizus* chitosan (5.2 kDa) described by [37]. No other articles were found that evaluated the molecular mass of chitosan from fungi of *Rhizopus*, except for *Rhizopus oryzae*. However, the chitosan of this fungus is much larger than that of *R. arrhizus*. Different authors reported molecular mass values greater than 100 kDa [64,65], 200 kDa [66], and 300 kDa [62] for the chitosan from *R. oryzae*. This small molecular mass of chitosan from *R. arrhizus* is considered a positive characteristic as it allows it to be used in applications different from those with a high molecular mass, thereby preventing any possible market competition with high molecular mass chitosan.

The Chit-FGal sample had an apparent molecular mass of 3.6 kDa, representing a yield rate of 200 mg of GA/g of the sample. This increase in mass after the conjugation process with GA was approximately 50% higher than those obtained by [67,68], which used high molecular weight animal chitosan to obtain a conjugation yield with GA of 118 mg of GA/g and 128 mg GA/g, respectively. This greater conjugation of GA molecules with Chit-FGal may be related to their molecular masses. According to Liu et al. (2013), high molecular mass chitosan tends to give more viscous solutions, which makes it difficult for the H_2_O_2_ to attack chitosan and inhibits the formation of binding points for GA [68]. Therefore, the larger the chitosan, the less efficient this process is and vice versa.

#### 3.2.3. FTIR Spectrum of Chit-F and Chit-FGal

**Figure 3:**
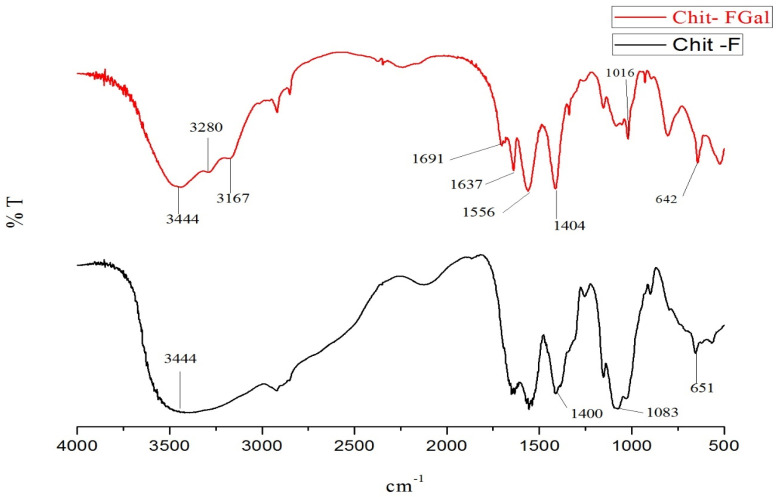
Fourier transformed infrared spectroscopy (FTIR). Chit-F (black) and fungal chitosan-gallic acid (Chit-FGal) (red) spectra. These spectra are representative of three independently performed analyses.

The infrared spectra of native chitosan (Chit-F) and gallic acid conjugated chitosan (Chit-FGal) are shown in Figure 3.

The infrared spectrum of both Chit-F and Chit-FGal presented bands found in chitosan [69,70]. The bands in the regions 642 cm^−1^ and 651 cm^−1^ indicate the presence of NH_2_ groups, whereas those in 1016 cm^−1^ and 1083 cm^−1^ represent the stretches of the CO bonding, and those between 1400 cm^−1^ and 1404 cm^−1^ represents the folding of CH_2_. The bands observed in 3167 cm^−1^, 3280 cm^−1^, and 3444 cm^−1^ represent NH and OH stretching and intramolecular hydrogen bonds of the water molecules of the solvation layer and bands described in other papers [71,72,73,74].

Chit-FGal presented two different bands, which indicate the covalent binding of GA to the native chitosan molecule. In the 1556 cm^−1^ region, there is a band indicating the presence of a C=O group of the amide formed when GA binds to the NH_2_ region. In the region between 1637 cm^−1^ e 1691 cm^−1^, the bands indicate the stretching of the C=O of the secondary amide formed when GA was covalently bound to the amino group. These bands appear only in the Chit-FGal spectrum, showing that there was a modification of the chitosan sample due to its covalent bond with GA. These bands that indicate the presence of GA were also described by other authors who promoted the conjugation of GA with animal chitosan [41] and beta-glucan [75], which confirms the conjugation of GA with fungal chitosan.

#### 3.2.4. NMR Analysis

**Figure 4:**
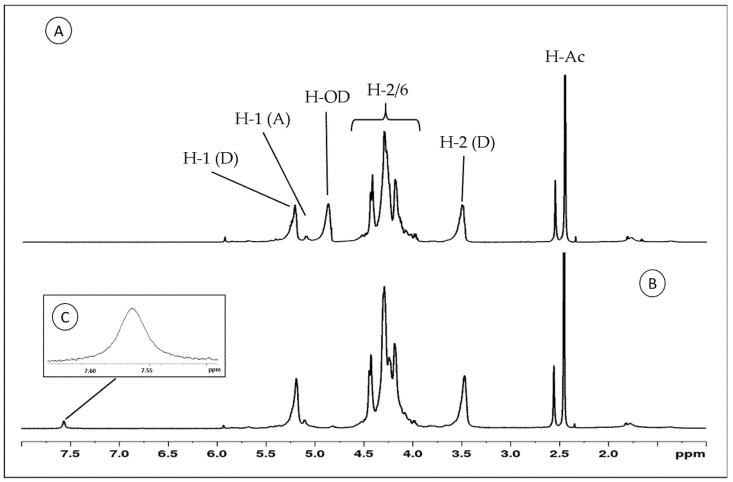
Proton-nuclear magnetic resonance (^1^H-NMR) spectra of Chit-F and Chit-FGal. (**A**) Chit-F spectra (**B**) Chit-FGal spectra (**C**) 7.57 Chit-FGal sign highlighted indicates regions where aromatic compounds are found. These spectra are representative of three independently performed analyses.

The samples were subjected to NMR analysis, and the ^1^H-NMR spectra of Chit-F and Chit-FGal are shown in Figure 4.

Both samples show typical signs of chitosan [41,76,77,78]. In the region ranged from 3.90 to 4.45 ppm, the signals referring to the hydrogens 2, 3, 4, 5, and 6 of the aldohexoses overlap, and therefore it was impossible to identify them separately. On the other hand, the signals of the two anomeric hydrogens of 2-amino-2-deoxy-D-glucopyranose (glucosamine) and 2-acetamido-2-deoxy-D-glucopyranose (N-acetyl-glucosamine) at 5.25 and 5.01 ppm, respectively, are clearly identified. In addition, the signal at 3.56 ppm corresponds to glucosamine H2 (D), and at 2.45 ppm, there is a signal from methyl group of N-acetyl-glucosamine (H-AC).

The 7.57 signal was found only in the Chit-FGal spectrum. According to Queiroz et al. [41], signals between 7.00 and 9.00 ppm indicate regions where aromatic compounds are found, which indicates a covalent bond between chitosan and GA. This signal has also been described in other polysaccharides that have been conjugated to GA, such as animal chitosan [41,68,79] and glucans [75].

#### 3.2.5. SEM Analysis

**Figure 5:**
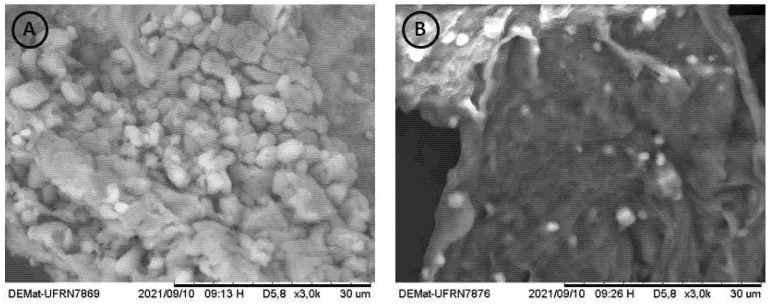
Microbiological biopolymers from R. arrizhus URM 8111—SEM electromicrographies of (**A**) Chit-F at 3000× magnification, (**B**) Chit-FGal at 3000× magnification. The measurement bar = 30 µm. Each short section corresponds to 3 µm.

The SEM images of the chitosans are shown in Figure 5. It is possible to observe that the Chit-F structure is less dense and compact, with an accentuated presence of pores and flake aggregates. Chit-FGal, on the other hand, presented a denser and more compact structure, a result similar to that found by Berger et al. [37] when performing SEM on chitosan extracted from Cunninghamella elegans.

As already reported in the literature, fungal chitosans did not show the microfibrillar structure in SEM, thus differentiating itself from chitin [80]. Both Chit-F and Chit-FGal did not show microfibrillar structure, confirming once more that it is chitosan and that modification occurred.

### 3.3. In Vitro Antioxidant Activity

#### 3.3.1. Copper Chelating Activity

**Figure 6:**
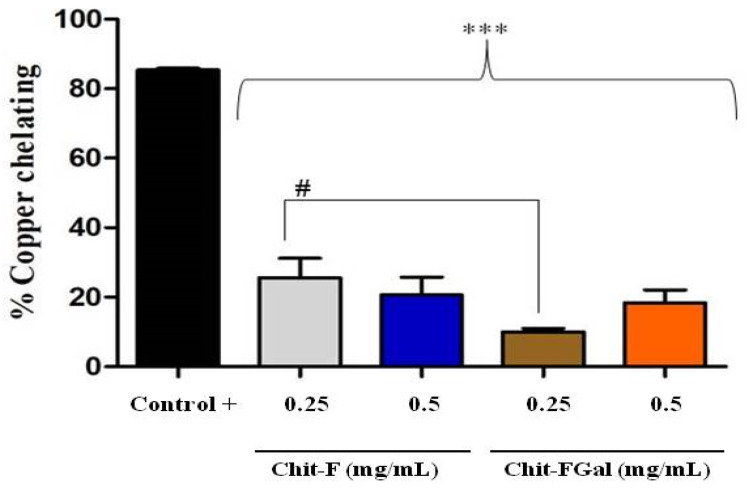
Chit-F and Chit-FGal copper chelation. The copper chelation tests were performed with GA at a concentration of 0.5 mg/mL, which did not show copper chelation activity under the evaluated conditions. *** indicates the significant difference between the controls and samples (*p* < 0.05). # indicates the significant difference between the same concentrations of different samples (*p* < 0.05).

Figure 6 shows the result of the copper chelating capacity of Chit-F and Chit-FGal. Both Chit-F and Chit-FGal (0.5 mg/mL) demonstrated low copper chelating capacity, approximately 20%, which shows that the conjugation did not enhance the chelating activity of the fungal chitosan. In fact, at the concentration of 0.25 mg/mL, the presence of GA caused the chelating activity of Chit-FGal to be significantly lower than that of Chit-F. This behavior has already been reported [41]. When animal chitosan was conjugated with GA, we observed significantly lower copper chelating activity, at low concentrations (from 0.015 to 0.25 mg/mL), than unconjugated chitosan. However, at higher concentrations (from 0.5 mg/mL), there was no significant difference between the activities of the two chitosan [41]. Previous data indicate an affinity of copper with chitosan due to the amine and hydroxyl (OH) grouping of carbon 3 [81,82,83]. As these two groups are entry points for GA (Figure 1), they are in less quantity in Chit-FGal, which justifies their lower chelating activity. However, when there was an increase in Chit-FGal, these deficiencies are overcome, and Chit-FGal showed activity similar to that of Chit-F.

#### 3.3.2. Total Antioxidant Capacity (TAC)

**Figure 7:**
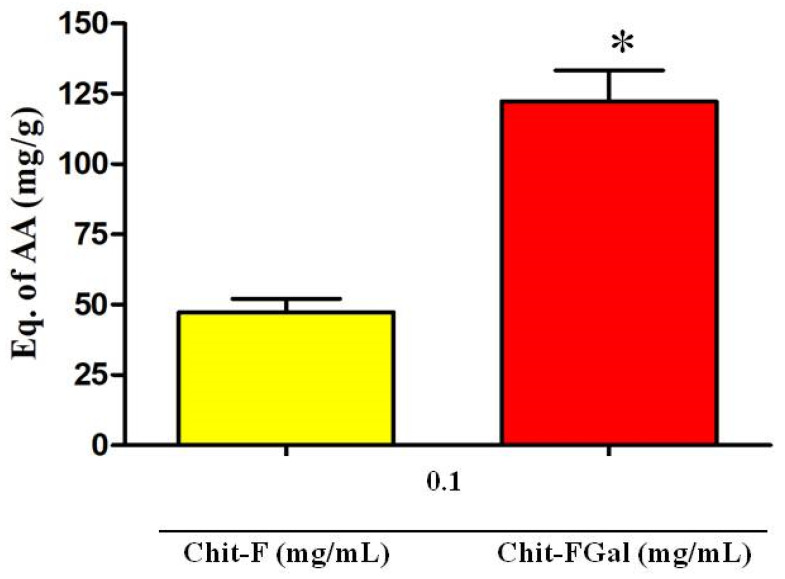
Total antioxidant capacity (TAC) test of Chit-F and Chit-FGal. * indicates significant difference between the samples (*p* < 0.05).

The TAC test assesses the ability of the sample to donate electrons to a reactive species. The greater this capacity, the greater is the probability of the sample to combat oxidative damage caused by reactive species. As shown in Figure 5, Chit-F had total antioxidant activity equivalent to 50 mg of ascorbic acid (AA)/g of sample, which means that 1 g of native Chit-F has the same activity as 50 mg of AA, the standard test substance. When conjugated with gallic acid (Chit-FGal sample), the total antioxidant capacity of chitosan increased approximately 3-fold to 125 mg AA/g sample (Figure 7).

This increase in Chit-FGal activity appears to be related to the amount of GA inserted into the molecule. This idea corroborates the finding that the GA-conjugated animal chitosan synthesized by [50], which had 7 mg GA per g of Chit, showed TAC of 5 mg AA/g. However, additional data referring to other GA-conjugated chitosan may confirm this observation.

### 3.4. Evaluation of Chit-FGal Antioxidant Activity under Different Cell Culture Conditions

As observed in the TAC assay, Chit-FGal had higher antioxidant activity than Quit-F. To confirm whether this activity was also observed in more complex conditions, we choose a model of culture of cells under stress conditions. Stress conditions can be introduced in the culture by the addition of various reagents, such as iron sulfate, copper sulfate [51], and H_2_O_2_ [54]. As Quit-FGal was much more effective in the test that assesses the electron-donating capacity (TAC) than the iron chelation test, we chose to induce cell stress with H_2_O_2_ and evaluate the cytoprotective capacity of Chit-FGal against this stressful condition.

**Figure 8:**
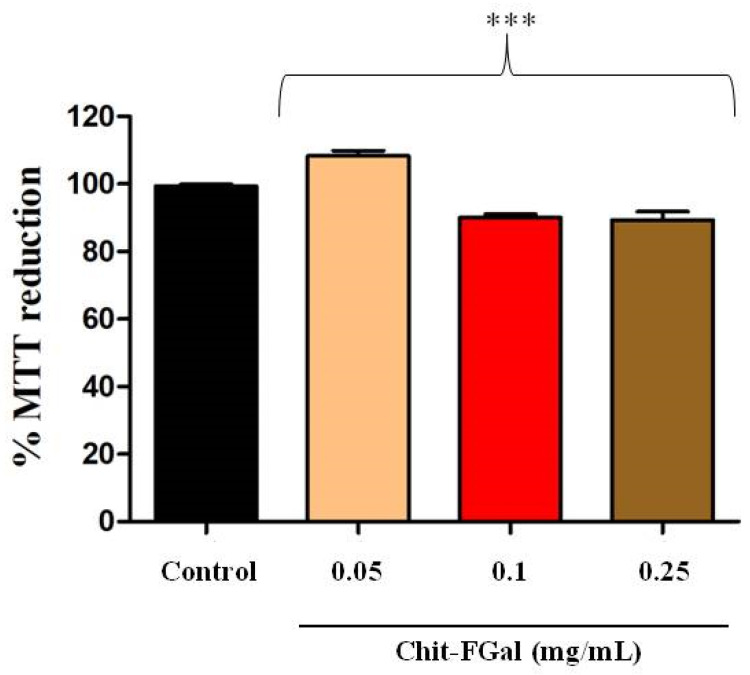
3-(4,5-Dimethylthiazol-2-yl)-2,5-diphenyltetrazolium bromide (MTT) reducing activity of the 3T3 fibroblast cells incubated with Chit-FGal for 24 h. Values are expressed as the mean ± standard deviation at concentrations of 0.05, 0.1, and 0.25 mg/mL. *** indicates the significant difference between the control and samples (*p* < 0.05).

Initially, to exclude any toxic effect of Chit-FGal, the cells were incubated with different concentrations of this chitosan for 24 h. In Figure 8, it is possible to observe that the cytoprotective capacity of the 3T3 cells treated with Chit-FGal was not affected in any of the conditions evaluated. This result indicates that Chit-FGal has no cytotoxic action.

**Figure 9:**
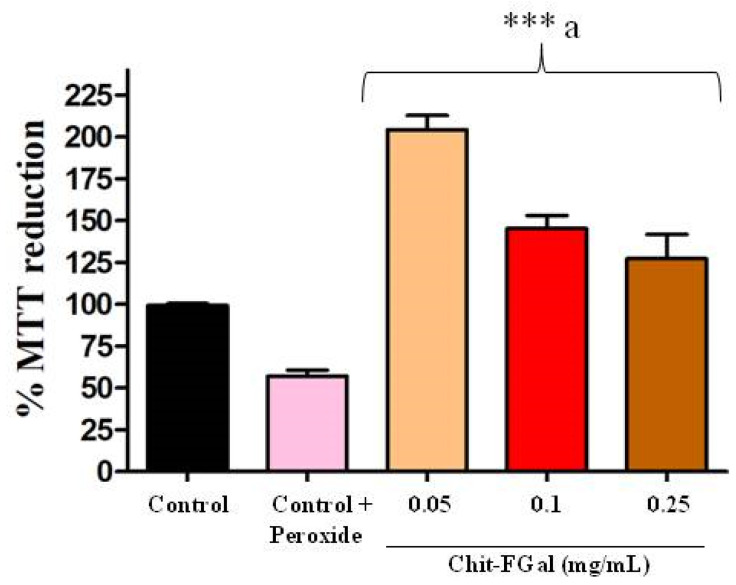
MTT reducing activity of the 3T3 fibroblast cells incubated with Chit-FGal and hydrogen peroxide (H_2_O_2_) for 6 h. Values are expressed as the mean ± standard deviation at concentrations of 0.05, 0.1, and 0.25 mg/mL. *** indicates the significant difference between the control and samples (*p* < 0.05). ^a^ indicates significant difference between the control + peroxide and the tested samples (*p* < 0.05).

As Chit-FGal did not show any cytotoxicity, we evaluated its ability to protect cells from oxidative damage caused by H_2_O_2_ (See Figure 9). We found that H_2_O_2_ (0.06 M) promoted a 50% decrease in the abilities of these cells to reduce MTT, which indicates cytotoxicity. However, the cells exposed to Chit-FGal, regardless of concentration, reduced MTT significantly more than the cells that were not exposed to peroxide. This result indicates that Chit-FGal protected the cells from oxidative damage.

When cells were exposed to Chit-FGal (0.05 mg/mL), their ability to reduce MTT was twice more than that observed in the control group. Chit-FGal (0.05 mg/mL) also promoted a greater reduction in MTT (See Figure 9). The data suggest that a low concentration of Chit-FGal activates the intracellular oxide reductase enzymes and/or stimulates the proliferation of cells. However, this needs to be assessed further in future studies.

**Figure 10:**
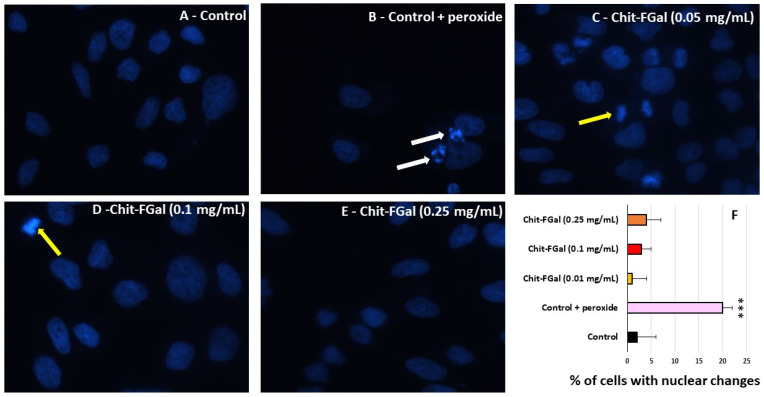
Morphological changes of 3T3 cells incubated with Chit-FGal and hydrogen peroxide (H_2_O_2_) for 6 h followed by DAPI staining. (**A**) Fluorescence microscope photographs of untreated cells; (**B**) cells treated with H_2_O_2;_ (**C**) cells treated with 0.05 mg/mL Chit-FGal + H_2_O_2_; (**D**) cells treated with 0.1 mg/mL Chit-FGal + H_2_O_2_; (**E**) cells treated with 0.25 mg/mL Chit-FGal + H_2_O_2_; (**F**) DAPI staining quantification—Images were captured from ten different fields and about 100 cells were counted for each field. White arrows indicate nuclear fragmentation and/or chromatin condensation. Yellow arrows indicate cell division. Magnification ×400. *** indicates the significant difference between the control and samples (*p* < 0.05).

To confirm the effect of Chit-FGal in the MTT tests, 3T3 cells were exposed to stress conditions in the presence of Chit-FGal, and nuclear DAPI staining was performed. As shown in Figure 10, nuclei with condensed chromatin of different sizes containing well-preserved but compacted cytoplasmic organelles and/or nuclear fragments were observed in H_2_O_2_ treated 3T3 cells, whereas the cells treated with different amounts of Chit-FGal did not show these morphological alterations. The cells were counted and the percentage of cells with chromatin condensation or nuclear fragmentation in each image (see the Experimental Section) was used to produce Figure 10F. The findings showed that Chit-FGal protects cell nuclei from damage caused by hydrogen peroxide.

According to Gutteridge and Halliwell 2010, the events of ROS generation via the Fenton and Haber–Weiss reactions start with the accumulation of superoxide and H_2_O_2_, resulting in a chemical imbalance and leading to the formation of more products. The interaction of peroxide with Fe^2+^ (mainly) or Cu^+^ leads to the formation of the hydroxyl radical (OH^−^) [84], which can cause significant damage to organisms, eventually resulting in various diseases such as cancer, diabetes, and Parkinson’s disease as well as aging. Therefore, by exhibiting copper chelating activity and TAC, in addition to protecting cells against stress caused by H_2_O_2_, Chit-FGal shows potential in decreasing the generation of ROS, thereby preventing excessive damage, and decreasing the risk of various diseases.

## 4. Conclusions

In the present study, we successfully isolated chitosan (Chit-F) from the fungus, *R. arrhizus*. The isolated Chit-F possessed a low molecular weight and a high degree of deacetylation and was free of phenolic compounds and proteins. We also demonstrated the possibility of chemically modifying this fungal chitosan molecule via a redox system with H_2_O_2_ and ascorbic acid to form Chit-FGal. This novel chitosan molecule (Chit-FGal) exhibited an antioxidant activity (ability to donate electrons) that was twice as high as that of the unmodified chitosan. Furthermore, Chit-FGal was able to protect 3T3 fibroblast cells from oxidative stress caused by H_2_O_2_. In summary, the results of our study indicate that Chit-FGal may be a promising molecule to combat oxidative stress.

We intend in the future to make a better structural characterization of Chit-FGal, including indicating the places where the gallic acid is covalently linked to the molecule. In addition, we intend to evaluate Chit-FGal as an antioxidant agent using different in vivo models, as well as to identify its antioxidant action mechanism.

## Figures and Tables

**Table 1:** Quantification of total proteins and phenolic compounds and determination of their apparent molecular weights.

	Protein (%)	Phenolics Compounds (%)	Molecular Weight (kDa)
Chit-F	nd	nd	3.0 ± 0.01
Chit-FGal	nd	4.0 ± 0.01	3.6 ± 0.03
